# Pilot Study of an Alpha-2-Macroglobulin-Enriched Plasma-Derived Orthobiologic Preparation in Sport Horses with Chronic Degenerative Joint Disease

**DOI:** 10.3390/vetsci13060536

**Published:** 2026-05-29

**Authors:** Enrico Gugliandolo, Vito Biondi, Maria De Luca, Elena Nangano, Giorgio Strozzi, Francesco Tosto, Gianluca Antonio Franco, Yanne Van Reusel, Giuseppe Catone, Jan H. Spaas

**Affiliations:** 1 Department of Veterinary Sciences, University of Messina, 98168 Messina, Italy; vito.biondi@unime.it (V.B.); mari.deluca.96@gmail.com (M.D.L.); elena.nangano1@studenti.unime.it (E.N.); gianluca.franco@studenti.unime.it (G.A.F.); gcatone@unime.it (G.C.); 2 Veterinary Consultant (Freelance), I98166 Messina, taly; giorgiostrozzi@gmail.com (G.S.); tostodotf@libero.it (F.T.); 3 Department of Research and Development, INTIBIO, 3960 Bree, Belgium; yanne@intibio.com (Y.V.R.); jan@intibio.com (J.H.S.); 4 Department of Morphology, Imaging, Orthopedics, Rehabilitation and Nutrition, Faculty of Veterinary Medicine, Ghent University, 9820 Merelbeke, Belgium

**Keywords:** alpha-2-macroglobulin, equine osteoarthritis, synovial fluid, lameness, intra-articular therapy, biomarkers, matrix metalloproteinases, inflammation, sport horses, pilot study

## Abstract

Chronic joint disease is a major cause of lameness and reduced performance in sport horses and is characterized by persistent synovial inflammation and protease-mediated matrix degradation. This exploratory prospective pilot study investigated clinical outcomes and synovial biomarker changes following intra-articular administration of an α-2-macroglobulin plasma-derived preparation. Twenty client-owned sport horses were observed in the treatment group (n = 10) or a comparison group (n = 10) and monitored for up to 180 days under field conditions. Clinical outcomes were assessed longitudinally, while synovial fluid was analyzed at baseline and 30 days post-treatment only in treated horses. Mixed-effects analysis showed significant group × time interactions for American Association of Equine Practitioners (AAEP) lameness score, flexion test response, and joint effusion. Treated horses showed early and sustained improvement in clinical scores, whereas minimal changes were observed in the comparison group. At 30 days, treated horses exhibited consistent within-subject reductions in synovial total protein, total nucleated cell count, polynuclear cell percentage, pro-inflammatory cytokines (PGE_2_, TNF-α, IL-6, IL-1β), matrix metalloproteinases (MMP-9, MMP-13), sulphated glycosaminoglycans, and neurogenic mediators (NGF, Substance P). These findings indicate a coherent pattern of clinical improvement associated with parallel changes in synovial biomarkers in treated horses. However, as longitudinal biomarker data were not collected in the comparison group, these observations should be interpreted as exploratory and do not establish causality. The observed findings support the rationale for further investigation of protease-targeted approaches in equine joint disease.

## Simple Summary

Chronic joint disease is a common cause of pain, lameness, and reduced athletic performance in sport horses. Current treatments mainly focus on symptom control and may not fully address the inflammatory and degradative processes associated with joint deterioration. In this observational prospective pilot study, ten client-owned sport horses with chronic joint disease were followed for up to six months under field conditions. Horses received a single intra-articular administration of an α-2-macroglobulin plasma-derived orthobiologic preparation. Clinical follow-up included lameness evaluation, flexion test response, joint effusion assessment, and overall functional scoring. In treated horses, synovial fluid was also analyzed before treatment and 30 days later to evaluate biomarkers associated with inflammation, tissue degradation, and pain. Treated horses showed improvement in several clinical parameters over time. In addition, treated horses demonstrated within-subject reductions in multiple synovial biomarkers associated with inflammation and cartilage catabolism. However, because longitudinal biomarker analyses were not performed in comparison horses and the study was not randomized, these findings should be interpreted as exploratory and hypothesis-generating rather than as definitive evidence of treatment-specific effects. Further randomized controlled studies with larger and more homogeneous populations are needed to better define the biological and clinical relevance of this orthobiologic approach in equine joint disease.

## 1. Introduction

Chronic joint disease represents one of the leading causes of lameness and reduced athletic performance in sport horses [1]. Synovitis and early osteoarthritic changes are commonly identified in actively competing animals and may progress despite conventional intra-articular therapies primarily aimed at short-term symptom control. Although current treatments can alleviate inflammation and improve clinical signs, sustained modulation of the intra-articular microenvironment remains a therapeutic challenge [2]. Current intra-articular therapies used in equine sports medicine primarily aim to control synovial inflammation and pain. Corticosteroids remain widely used for short-term symptom control [3], whereas biologic therapies such as platelet-rich plasma (PRP), autologous conditioned serum (IRAP), and mesenchymal stromal cell therapies have been introduced with the aim of modulating inflammatory pathways and supporting joint homeostasis [1]. While these approaches may provide clinical benefit, their mechanisms of action largely target inflammatory signaling rather than the broader proteolytic cascades responsible for extracellular matrix degradation. Increasing evidence indicates that osteoarthritis is not solely a mechanical or inflammatory disorder but a complex process driven by interconnected inflammatory and proteolytic pathways [4]. Matrix metalloproteinases (MMPs) and other proteases play a central role in extracellular matrix degradation and cartilage collagen breakdown [5]. In particular, MMP-3, MMP-9 and MMP-13 contribute to proteoglycan loss and collagen degradation, thereby amplifying synovial inflammation and accelerating cartilage deterioration. Pro-inflammatory cytokines such as IL-1β, TNF-α and IL-6 further perpetuate this catabolic cascade [6], while mediators including nerve growth factor (NGF) and Substance P contribute to nociceptive sensitization within the joint [7]. The resulting imbalance between anabolic and catabolic processes promotes progressive structural and functional deterioration. Targeting protease-driven mechanisms has therefore emerged as a potential strategy to more comprehensively modulate the joint microenvironment. Alpha-2-macroglobulin (A2MG) is a broad-spectrum endogenous protease inhibitor capable of binding and neutralizing multiple classes of activated proteases, including matrix metalloproteinases and inflammatory mediators [8]. By acting as a molecular “trap” A2MG may attenuate upstream drivers of proteolytic and inflammatory signaling rather than targeting isolated downstream mediators [9]. Experimental and translational evidence supports the biological plausibility of protease inhibition in joint disease [10,11,12]. However, clinical evidence evaluating the effects of protease-targeted therapies on both clinical outcomes and synovial molecular pathways in naturally occurring equine joint disease remains limited. Most available studies have focused on short-term clinical responses, with limited integration of synovial biomarker analysis and longitudinal functional assessment. A more comprehensive evaluation incorporating clinical scoring, synovial inflammatory and proteolytic markers, and real-world functional outcomes may therefore help clarify the biological coherence of protease-targeted strategies in sport horses. The aim of this prospective controlled pilot study was therefore to evaluate clinical outcomes and synovial biomarker modulation following intra-articular alpha-2-macroglobulin therapy in sport horses affected by chronic joint disease. Recent equine orthobiologic characterization studies have demonstrated that PRP- and A2MG-based formulations exhibit distinct but complementary biochemical profiles involving growth factors, anabolic mediators, and immunomodulatory components [13]. Accordingly, the biological effects of plasma-derived orthobiologics likely reflect the interaction of multiple bioactive mediators within the joint microenvironment. We hypothesized that treatment would result in sustained clinical improvement accompanied by coordinated modulation of inflammatory, proteolytic and neurogenic mediators within the synovial microenvironment.

## 2. Materials and Methods

### 2.1. Study Design

Twenty client-owned jumping horses presenting with persistent lameness, joint pain during orthopedic examination and flexion testing, joint effusion, and imaging findings consistent with chronic degenerative joint pathology affecting high-motion joints were enrolled as shown in Table 1 Clinical signs persisted for more than four weeks despite routine clinical management. All enrolled horses had a previous history of partial clinical response to non-steroidal anti-inflammatory drugs (NSAIDs), characterized by transient improvement without complete or sustained clinical resolution. To minimize pharmacological interference, all horses underwent a predefined washout period before enrolment. No systemic anti-inflammatory, analgesic, or intra-articular pharmacological treatments were administered during the study follow-up period. Horses were allocated in a 1:1 ratio to either a treatment group (A2MG, n = 10) or a comparison group (CTR, n = 10). Group allocation was based on clinical decision-making and owner consent, reflecting routine clinical management conditions commonly encountered in equine sports medicine practice. Horses whose owners elected intra-articular treatment were assigned to the A2MG group, whereas horses managed conservatively were included in the comparison group. Conservative management consisted of controlled exercise modulation and routine clinical monitoring without systemic anti-inflammatory, analgesic, or intra-articular pharmacological treatment during the study period. No formal randomization procedure was applied. Horses receiving systemic anti-inflammatory or analgesic medications within the predefined washout period before enrolment were excluded. Control horses did not receive intra-articular therapy during the study period but were managed under the same standardized clinical monitoring and exercise conditions. Clinical outcomes were assessed at baseline (T0) and at 7 (T7), 30 (T30), 120 (T120), and 180 (T180) days and included AAEP lameness grading, flexion test response, joint effusion scoring, and Total Functional Score evaluation. The exercise regimen was standardized as much as possible under field conditions and consisted of daily ridden activity including walking, trotting, and canter exercise, with limited jumping activity performed approximately once weekly according to each horse’s clinical condition and training status. Horses were not maintained under complete stall rest or training suspension during follow-up.

Synovial fluid was collected at T0 and T30 exclusively from horses in the A2MG-treated group for exploratory biomarker analysis. Repeat arthrocentesis was not performed in comparison horses in order to avoid invasive joint procedures without direct therapeutic indication under field clinical conditions. The comparison group was included to provide a clinical reference for longitudinal changes in lameness, flexion response, joint effusion, and functional outcomes over the same follow-up period. It was not intended for longitudinal synovial biomarker comparison. Therefore, the primary objective of the study was to explore longitudinal clinical trajectories between treated and comparison horses, whereas the secondary exploratory objective was to describe within-subject synovial biomarker changes in treated horses only. The study was conducted in accordance with institutional guidelines for clinical research and approved by the Ethical Committee of the University of Messina (10/2025bis) and Virtus Bonarum Cellarum (EC_2024_001, 2024). Inclusion of multiple joint types (fetlock, tarsal, and stifle joints) was intended to reflect the heterogeneous clinical presentation commonly encountered in equine sport-related degenerative joint disease under field conditions.

**Table 1:** Demographic and clinical characteristics of horses included in the study.

Comparison Group				
Horse	Age	kg	Discipline	Joint
1	9	480	jumping	Stifle
2	9	430	Jumping	Stifle
3	11	455	Jumping	Fetlock
4	12	490	Jumping	Fetlock
5	11	390	Jumping	Fetlock
6	8	410	Jumping	Fetlock
7	7	500	Jumping	Tarsus
8	13	520	Jumping	Tarsus
9	14	480	Jumping	Tarsus
10	13	470	jumping	Tarsus
A2MG Group				
Horse	Age	kg	Discipline	Joint
1	7	350	jumping	Stifle
2	10	450	Jumping	Fetlock
3	8	400	Jumping	Fetlock
4	12	480	Jumping	Fetlock
5	13	500	Jumping	Stifle
6	9	490	Jumping	Tarsus
7	14	450	Jumping	Tarsus
8	11	510	Jumping	Fetlock
9	12	400	Jumping	Tarsus
10	13	470	jumping	Tarsus

### 2.2. Inclusion and Exclusion Criteria

Horses were eligible if they were actively trained sport horses presenting with chronic (≥4 weeks) unilateral lameness or reduced performance attributable to a single joint, associated with clinical evidence of synovial effusion and imaging findings consistent with chronic degenerative joint pathology.

Inclusion required a positive flexion test of the affected joint; when indicated, improvement following regional diagnostic anesthesia; and radiographic and/or ultrasonographic evidence of degenerative joint changes.

Exclusion criteria included clinical or laboratory suspicion of septic arthritis; acute traumatic joint injury; intra-articular therapy within the previous 8 weeks; systemic anti-inflammatory treatment within 7 days prior to baseline; concurrent orthopedic conditions affecting other limbs; systemic disease potentially interfering with inflammatory biomarkers; or a traumatic joint event.

### 2.3. Synovial Fluid Sampling and Intraarticular Therapy Procedure

All procedures were performed under standardized aseptic conditions. The affected joint was clipped and surgically prepared using chlorhexidine scrub followed by alcohol. At baseline (T0), a minimum of 2 mL of synovial fluid was aspirated prior to treatment. Immediately thereafter, A2MG-enriched plasma-derived orthobiologic preparation (A2MG Plus, INTIBIO, Bree, Belgium) was performed according to the manufacturer’s recommended protocol. Briefly, blood was taken in citrate and centrifuged at 2160× *g* for 10 min. The plasma was brought in filter tubes containing a conical 100 micron filter and centrifuged at 2160× *g* for 10 min, allowing the larger blood proteins to stay on top of the filter according to the manufacturer’s system (A2MG Plus, INTIBIO, Belgium). The plasma was subsequently aliquoted at a volume of 4 mL or 6 mL per sterile vials. A volume of 4 mL was injected for fetlock and tarsal joints, and 6 mL for stifle joints, reflecting joint-specific administration guidelines. No intra-articular local anesthetics were used, and no sedation was administered during the procedure. Following injection, horses were monitored for immediate adverse reactions and returned to controlled activity according to clinical recommendations. No additional intra-articular therapies were administered during the follow-up period. At 30 days post-treatment (T30), repeat arthrocentesis was performed under identical aseptic conditions for synovial fluid collection as part of the routine clinical follow-up and therapeutic management of the treated joint.

### 2.4. Synovial Fluid Processing and Biomarker Analysis

Synovial fluid samples were collected aseptically from the A2MG-treated horses only and immediately centrifuged at 3000× *g* for 10 min at 4 °C to remove cellular debris. Supernatants were visually inspected for blood contamination, aliquoted into sterile polypropylene tubes, and stored at −80 °C until batch analysis. Samples underwent a maximum of one freeze–thaw cycle prior to analysis. Laboratory investigators were blinded to time point during all analyses. Total nucleated cell count (TNCC) was determined using a manual hemacytometer. Differential cytology was assessed on preparations stained with May–Grünwald–Giemsa, and cell populations were expressed as percentage of total nucleated cells. Total protein concentration was quantified using a bicinchoninic acid (BCA) colorimetric assay (Thermo Fisher Scientific, Waltham, MA, USA) according to the manufacturer’s protocol. Samples were diluted 1:40 in phosphate-buffered saline (PBS) to ensure measurements within the linear dynamic range. Calibration curves were generated using serial dilutions of bovine serum albumin (BSA), with a minimum coefficient of determination (R^2^) of 0.99 required for acceptance. Absorbance was measured at 562 nm using a microplate reader, and protein concentrations were calculated by interpolation from the standard curve and expressed as mg/mL. Sulphated glycosaminoglycans (sGAGs) were quantified using the 1,9-dimethylmethylene blue (DMMB) dye-binding assay adapted for synovial fluid. Samples were diluted 1:30 in Milli-Q water and analyzed in duplicate. Chondroitin-4-sulfate standards (2.5–30 μg/mL) were used to generate calibration curves. Absorbance was recorded at 540 nm immediately after reagent addition to minimize dye–complex dissociation, and concentrations were expressed as μg/mL. Synovial concentrations of prostaglandin E2 (PGE2, Cat # CEA538Ge), tumor necrosis factor-α (TNF-α, Cat # SEA133Eq), interleukin-6 (IL-6, Cat # SEA079Eq), interleukin-1β (IL-1β, Cat # SEA563Eq), nerve growth factor (NGF, Cat # EK8F219), substance P (Cat # EEL013), matrix metalloproteinase-9 (MMP-9, Cat # SEA553Eq), and matrix metalloproteinase-13 (MMP-13, Cat # SEA099Eq) were quantified using commercially available enzyme-linked immunosorbent assay (ELISA) kits validated for equine samples or previously reported for use in equine synovial fluid. All ELISA measurements were performed in duplicate, and mean values were used for analysis. Results were accepted when the coefficient of variation (CV) between replicates was <15%. Assay performance characteristics, including intra- and inter-assay variability and detection limits, were consistent with the manufacturer’s specifications. Preliminary validation steps were performed to confirm assay suitability for equine synovial fluid, including assessment of dilution linearity within the working range. Values below the lower limit of detection were assigned the lowest detectable value for statistical analysis. To minimize inter-assay variability, samples from the same subject were analyzed within the same assay run whenever possible. All assays were performed within the validated dynamic range.

### 2.5. Clinical Assessment

Clinical evaluation was performed at each time point by two experienced investigators using predefined scoring criteria. Lameness was graded according to the American Association of Equine Practitioners (AAEP) 0–5 scale [14]. Horses were evaluated at the trot in hand in a straight line and on the lunge in both directions, on both hard and soft surfaces, to maximize detection of subtle gait abnormalities. The highest observed lameness grade during the examination was recorded as the final score. Treatment success was predefined as an improvement of at least one AAEP grade compared with baseline. A passive flexion test of the affected joint was performed by maintaining maximal joint flexion for 60 s without applying additional external force beyond physiological limits. Immediately following release, horses were trotted in a straight line, and the response was graded using a 5-point ordinal scale (0 = no response; 1 = a few lame steps; 2 = consistent lameness; 3 = exaggerated lameness; 4 = minimal weight-bearing) [15]. Joint effusion was assessed by visual inspection and digital palpation and graded using a 5-point ordinal scale (0 = no swelling; 1 = subtle swelling; 2 = moderate swelling; 3 = marked swelling; 4 = severe distension) [16]. Clinical lameness (AAEP), flexion response, and joint effusion scores were independently assessed by two experienced clinicians blinded to group allocation. Assessments were performed using a standardized examination protocol.

### 2.6. Rider-Reported Functional Assessment

Rider-reported outcomes were collected at each clinical visit (T0, T7, T30, T60, T120, and T180) using a standardized structured questionnaire administered immediately after riding. Riders were not blinded to clinical management, and these measures were therefore considered exploratory secondary outcomes intended to complement objective clinical assessments [17]. The questionnaire included six items scored on a 4-point ordinal scale (0 = Poor, 1 = Fair, 2 = Good, 3 = Excellent).

Items 1–3 assessed current functional status:

1. Comfort under saddle;
2. Overall quality of gaits;
3. Willingness and forwardness during warm-up.

A Total Functional Score (range 0–9) was calculated as the sum of items 1–3 at each time point.

Items 4–6 assessed perceived change relative to baseline (T0):

4. Perceived improvement in performance;
5. Recovery to full athletic potential;
6. Readiness to return to competition.

Statistical analyses were performed using GraphPad Prism 11 (GraphPad Software, San Diego, CA, USA).

### 2.7. Statistical Analysis

Statistical analyses were performed using GraphPad Prism version 10 (GraphPad Software, San Diego, CA, USA). An a priori sample size calculation was performed using G*Power v11 based on the primary clinical outcome, defined as the longitudinal change in AAEP lameness score. The calculation was based on a repeated-measures between–within interaction model with two groups and repeated assessments over time, assuming a two-sided α level of 0.05, a statistical power of 0.80, and a large, expected effect size. Based on these assumptions, the minimum required sample size was estimated at 10 horses per group. Repeated clinical outcomes (AAEP lameness grade, flexion score, joint effusion score, and Total Functional Score) were analyzed using a mixed-effects model with restricted maximum likelihood (REML) to account for repeated measures within individual horses. The model included group, time, and the group × time interaction as fixed effects, with horse included as a random effect. The primary statistical inference was based on the significance of the group × time interaction term, which evaluated whether temporal trajectories differed between treated and control horses. Where appropriate, the Geisser–Greenhouse correction was applied to adjust for violations of sphericity. Changes from baseline (Δ) at T30 were compared between groups using the two-tailed Mann–Whitney U test. Paired comparisons of synovial biomarkers between baseline (T0) and T30 in treated horses were analyzed using the Wilcoxon matched-pairs signed-rank test. All statistical tests were two-tailed, and statistical significance was set at *p* < 0.05. We acknowledge that ordinal models would be preferable for confirmatory inference. However, given the exploratory pilot design, small sample size, and longitudinal field setting, mixed-effects REML models were used to describe repeated-measure trajectories. The results are therefore interpreted as exploratory and hypothesis-generating.

## 3. Results

### 3.1. Clinical Outcome

**Figure 1:**
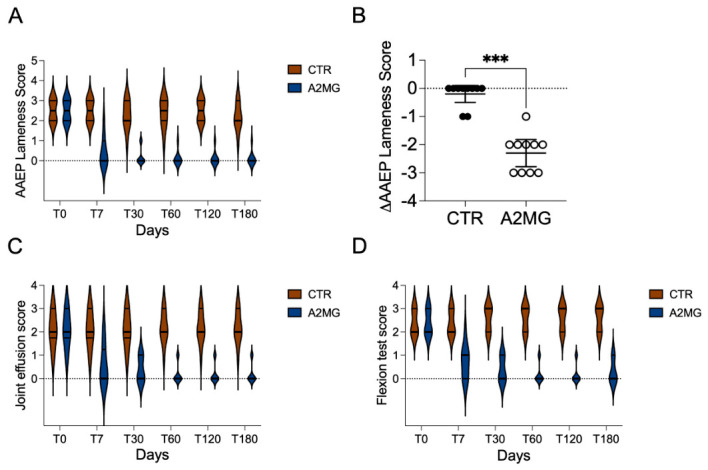
Longitudinal clinical outcomes following intra-articular A2MG administration. A2MG and control horses. Violin plots represent the distribution of AAEP lameness score (**A**), joint effusion score (**C**), and passive flexion test score (**D**). Central lines indicate median values. Mixed-effects model (REML) showed significant group × time interactions for all clinical parameters (*p* < 0.0001). (**B**) Change from baseline (ΔAAEP) at T30 in treated and control horses. Each point represents an individual horse. Horizontal lines indicate median and interquartile range. The reduction in lameness was significantly greater in the treated group compared with controls (Mann–Whitney test, exact *** *p* < 0.001).

No statistically significant differences were observed between groups at baseline in terms of age, weight, or distribution of affected joints. Mixed-effects modeling revealed a significant group × time interaction for AAEP lameness scores (F(2.718, 48.93), *p* < 0.0001), indicating divergent temporal trajectories between treated and control horses, as shown in Figure 1. The adjusted mean difference between groups across time points was 1.78 points (95% CI 1.35–2.21). Treated horses exhibited a rapid reduction in lameness beginning at T7, which was maintained through T120, whereas control horses showed minimal variation over time [8]. Comparable group × time interactions were observed for passive flexion test scores (F(2.382, 42.87), *p* < 0.0001) and joint effusion scores (F(2.397, 43.15), *p* < 0.0001), with adjusted mean differences of 1.74 (95% CI 1.31–2.17) and 1.44 (95% CI 0.87–2.01), respectively. At T30, the change from baseline (ΔAAEP) was significantly greater in treated horses compared with controls (Mann–Whitney U = 1, exact *p* < 0.001). Median change was 0 (IQR 0 to −1) in controls and −2 (IQR −3 to −2) in treated horses (Hodges–Lehmann estimate −2.0). No adverse effects were observed following intra-articular administration. At T7, 10/10 (100%) treated horses showed at least a one-grade improvement in AAEP lameness score, whereas none of the control horses showed clinical improvement (0/10).

**Figure 2:**
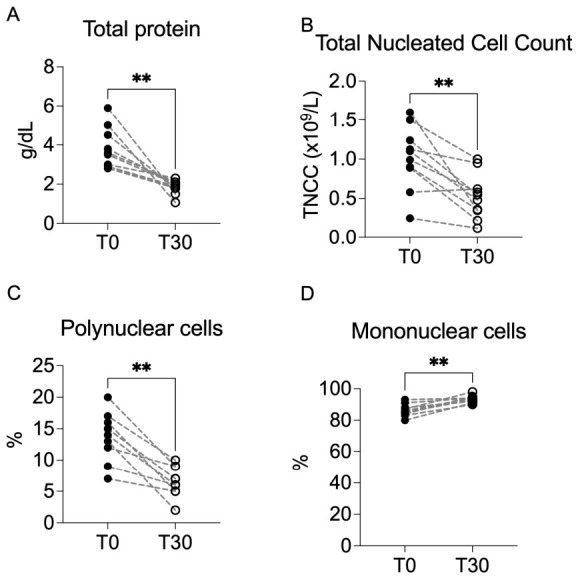
Modulation of synovial inflammatory profile at 30 days following A2MG treatment. Paired analysis of (**A**) total protein, (**B**) total nucleated cell count (TNCC), (**C**) polynuclear cells (%), and (**D**) mononuclear cells (%). Each line represents an individual horse. Data were analyzed using the Wilcoxon matched-pairs signed-rank test. Significant reductions were observed in total protein, TNCC, and polynuclear cells, with a reciprocal increase in mononuclear cells at T30. ** *p* < 0.01.

A consistent pattern of within-subject changes was observed 30 days after treatment. As shown in Figure 2 Total protein concentration significantly decreased from baseline to T30 (Wilcoxon matched-pairs signed-rank test, exact *p* < 0.001; median difference −1.50 g/dL). All treated horses showed a reduction in total protein at T30 compared with baseline. Total nucleated cell count (TNCC) was also significantly reduced at T30 (*p* = 0.004; median difference −0.52 × 10^9^/L). Differential cytology revealed a consistent shift in synovial cellular composition. The proportion of polynuclear cells significantly decreased (*p* = 0.002; median reduction −8 percentage points), while mononuclear cells increased correspondingly (*p* = 0.002; median increase +8 percentage points). All treated horses exhibited a reduction in polynuclear cell percentage and a reciprocal increase in mononuclear cells at T30. Collectively, these findings indicate a uniform shift in synovial cellular profile consistent with attenuation of inflammatory activity at 30 days.

### 3.2. Synovial Inflammatory Mediators

**Figure 3:**
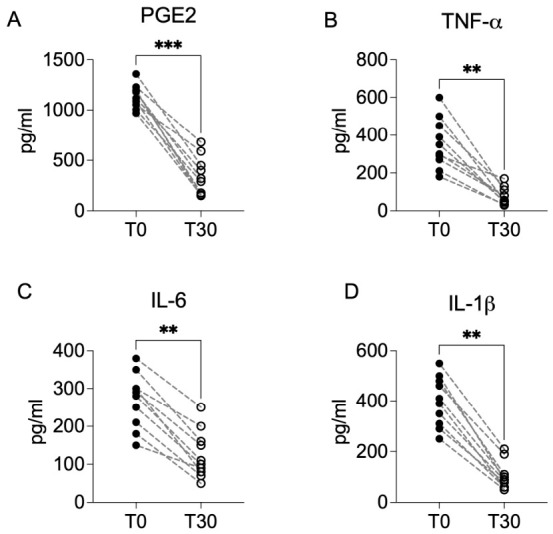
Significant reduction in synovial pro-inflammatory mediators at 30 days following A2MG treatment. Paired analysis of PGE2, TNF-α, IL-6, and IL-1β concentrations at baseline (T0) and T30. Individual paired values are shown. All analytes showed significant decreases at T30 compared with baseline (Wilcoxon matched-pairs signed-rank test). ** *p* < 0.01; *** *p* < 0.001.

As shown in Figure 3 thirty days after intra-articular administration, a coordinated reduction in synovial pro-inflammatory mediators was observed. Prostaglandin E2 (PGE2) concentrations markedly decreased at T30 compared with baseline (Wilcoxon matched-pairs signed-rank test, *p* < 0.001), representing the largest relative change among the analyzed mediators. All treated horses showed a reduction in PGE2 levels at T30. Tumor necrosis factor-α (TNF-α) concentrations were significantly reduced at T30 (*p* = 0.002), with consistent downward shifts across individual subjects. Similarly, interleukin-6 (IL-6) and interleukin-1β (IL-1β) concentrations significantly decreased from baseline (both *p* = 0.002), demonstrating a uniform reduction in inflammatory cytokine signaling within the treated joint. The consistent paired reductions across all evaluated mediators are consistent with a reduction in inflammatory activity at 30 days.

### 3.3. Cartilage Degradation Markers

**Figure 4:**
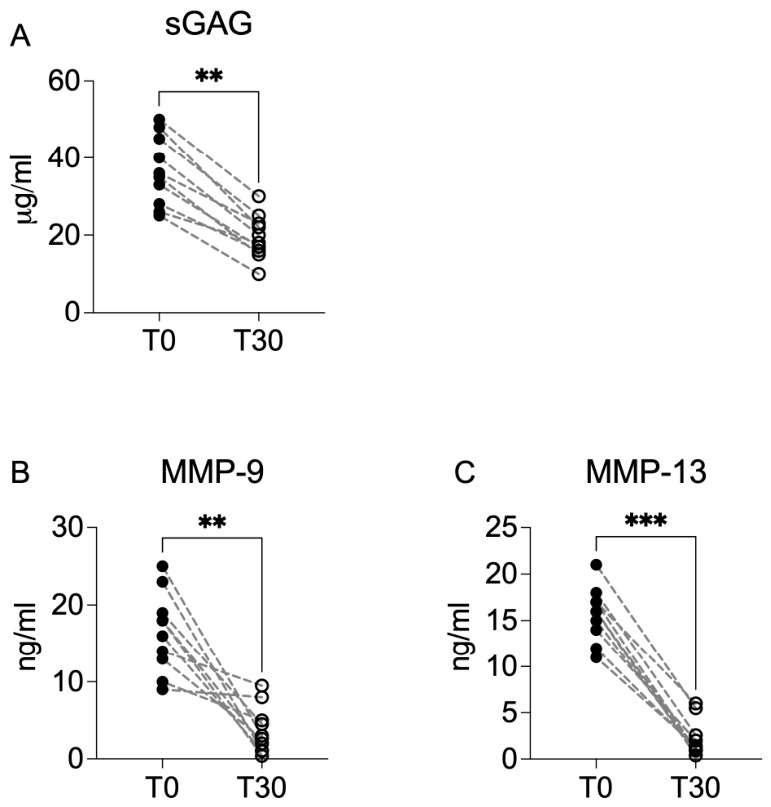
Reduction in synovial cartilage degradation markers following intra-articular A2MG administration. (**A**) Sulfated glycosaminoglycans (sGAG), (**B**) matrix metalloproteinase-9 (MMP-9), and (**C**) matrix metalloproteinase-13 (MMP-13) concentrations in synovial fluid at baseline (T0) and 30 days post-treatment (T30). Each line represents an individual horse (n = 10). Horizontal bars indicate median values. Statistical analysis was performed using the Wilcoxon matched-pairs signed-rank test (two-tailed). Significant reductions were observed for sGAG (*p* = 0.002), MMP-9 (*p* = 0.002), and MMP-13 (*p* < 0.001). ** *p* < 0.01; *** *p* < 0.001.

A coordinated reduction in synovial markers associated with extracellular matrix degradation was observed 30 days after intra-articular administration. Sulfated glycosaminoglycan (sGAG) concentrations significantly decreased at T30 compared with baseline (Wilcoxon matched-pairs signed-rank test, *p* = 0.002). All treated horses showed a reduction in sGAG levels at T30 relative to T0. Synovial matrix metalloproteinase-9 (MMP-9) levels were similarly reduced at T30 (*p* = 0.002), with consistent paired decreases across individual subjects. Matrix metalloproteinase-13 (MMP-13) exhibited the most pronounced reduction among the evaluated degradation markers (*p* < 0.001), with all treated horses showing marked decreases at T30. Collectively, these findings indicate a uniform attenuation of synovial catabolic mediator activity 30 days after treatment as shown in Figure 4.

### 3.4. Neurogenic Inflammatory Mediators

**Figure 5:**
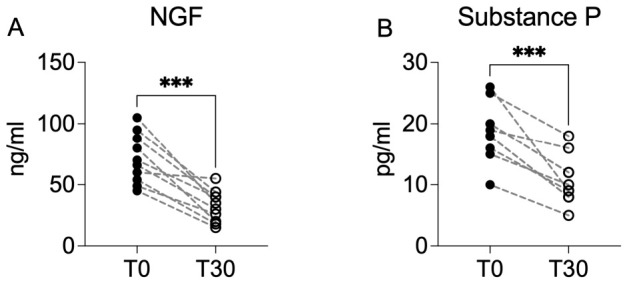
Reduction in synovial neurogenic mediators following intra-articular A2MG administration. (**A**) Nerve growth factor (NGF) and (**B**) Substance P concentrations in synovial fluid at baseline (T0) and 30 days post-treatment (T30). Each line represents an individual horse (n = 10). Horizontal bars indicate median values. Statistical analysis was performed using the Wilcoxon matched-pairs signed-rank test (two-tailed). Significant reductions were observed for NGF (*p* < 0.001) and Substance P (*p* < 0.001). *** *p* < 0.001.

A significant reduction in synovial mediators associated with neurogenic inflammatory signaling was observed 30 days after intra-articular administration. Nerve growth factor (NGF) concentrations significantly decreased at T30 compared with baseline (Wilcoxon matched-pairs signed-rank test, *p* < 0.001). All treated horses showed a reduction in NGF levels at 30 days. Similarly, synovial Substance P concentrations were significantly reduced at T30 (*p* < 0.001), with consistent paired decreases across individual subjects. As shown in Figure 5 The parallel reduction in NGF and Substance P indicates a modulation of synovial mediators implicated in nociceptive sensitization and neuro-inflammatory amplification within the joint microenvironment.

### 3.5. Rider-Reported Functional Outcomes

**Figure 6:**
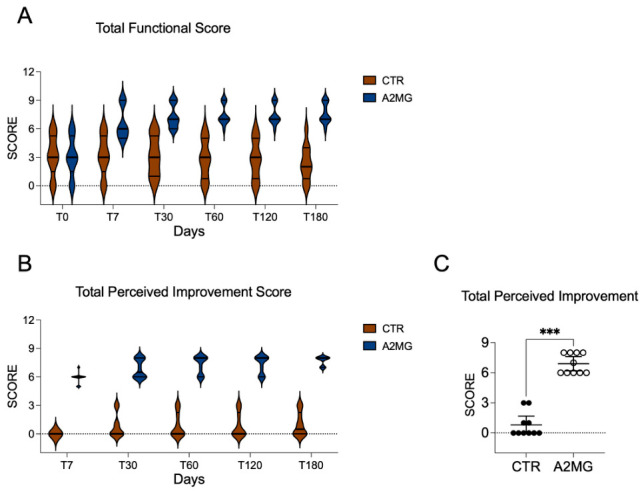
Rider-reported functional outcomes. (**A**) Longitudinal evolution of Total Functional Score (range 0–9; items 1–3) in treated (A2MG, n = 10) and control (CTR, n = 10) horses at T0, T7, T30, T60, T120, and T180. Violin plots represent score distribution; central lines indicate median values. Mixed-effects model showed a significant group × time interaction (*p* < 0.0001). (**B**) Total Perceived Improvement Score (range 0–9; items 4–6) at T30. Each point represents an individual horse; horizontal bars indicate median and interquartile range. (**C**) Change in total perceived improvment score Treated horses showed significantly higher perceived improvement compared with controls (Mann–Whitney test, exact *** *p* < 0.001). These exploratory outcomes were not blinded.

As shown in Figure 6 Mixed-effects analysis revealed a significant group × time interaction for Total Functional Score (*p* < 0.0001), indicating divergent longitudinal trajectories between treated and control horses. The adjusted mean difference across time points was −3.68 points (95% CI −5.31 to −2.06). Treated horses exhibited progressive improvement beginning at T7, which was sustained through T180, whereas control horses showed minimal variation over time. At T30, Total Perceived Improvement Scores were significantly higher in treated horses compared with controls (Mann–Whitney test, exact *p* < 0.001). Median scores were 7 (IQR 6–8) in treated horses and 0 (IQR 0–1) in controls. Scores remained stable during subsequent follow-up. Given the non-blinded nature of rider assessment, these findings are considered exploratory and supportive of the objective clinical outcomes.

## 4. Discussion

The present exploratory prospective pilot study showed that intra-articular administration of alpha-2-macroglobulin therapy was associated with sustained clinical improvement in sport horses affected by chronic joint disease, accompanied by coordinated modulation of synovial inflammatory, proteolytic and neurogenic mediators. The significant group × time interactions observed for all clinical parameters indicate clearly divergent temporal trajectories between treated and control horses, with rapid clinical improvement evident as early as seven days and maintained throughout the follow-up period. Alpha-2-macroglobulin is a large endogenous plasma protease inhibitor that functions as a broad-spectrum molecular “trap” for activated proteases [8]. Upon protease binding, A2MG undergoes a conformational change that entraps the enzyme and promotes its clearance through receptor-mediated pathways. In the context of joint disease, A2MG has been shown to inhibit multiple proteolytic enzymes implicated in cartilage degradation, including matrix metalloproteinases and other catabolic proteases, while also interacting with inflammatory mediators involved in synovial activation [10]. Experimental studies in osteoarthritis models have showed that intra-articular administration of A2MG can attenuate inflammatory signaling and reduce cartilage degradation [18]. On this basis, we hypothesized that intra-articular administration of A2MG in horses with chronic joint disease would modulate the synovial microenvironment by reducing protease activity and downstream inflammatory signaling pathways. Although A2MG enrichment represents a primary characteristic of the preparation used in this study, the investigated product remains a plasma-derived orthobiologic formulation containing multiple bioactive plasma-derived components that may contribute to the observed clinical and molecular changes. In this context, A2MG should be interpreted as a plausible mechanistic contributor within a broader multimodal biological framework rather than as the sole driver of the observed responses. Recent equine orthobiologic characterization studies from our group demonstrated that PRP- and A2MG-based orthobiologics possess distinct but complementary biochemical profiles, including differences in growth factors, anabolic mediators, and immunomodulatory components [13]. These findings support the concept that plasma-derived orthobiologic preparations may exert coordinated biological effects through multiple interacting pathways rather than through a single isolated mechanism.

In the present study, treated horses showed consistent clinical improvement over time (AAEP score). Although early responses in non-randomized designs should be interpreted with caution, the persistence of improvement through later time points suggests a pattern extending beyond short-term variability. In parallel, treated horses exhibited within-subject reductions in synovial biomarkers, including pro-inflammatory cytokines (PGE_2_, TNF-α, IL-6 and IL-1β), matrix metalloproteinases (MMP-9 and MMP-13), sulphated glycosaminoglycans, and neurogenic mediators (NGF and Substance P). These mediators are widely reported to be elevated in equine osteoarthritis and synovial inflammation [5,6], where they contribute to extracellular matrix degradation, inflammatory amplification, and nociceptive sensitization. Previous studies have shown that these biomarkers may remain elevated over time in affected joints, reflecting persistent activation of inflammatory and catabolic pathways [19]. Experimental and naturally occurring models of equine osteoarthritis have consistently shown increased concentrations of inflammatory cytokines, MMP-9, MMP-13 and cartilage degradation markers in synovial fluid during disease progression [5,6]. Moreover, several studies have shown that these mediators may remain elevated over prolonged periods in affected joints, reflecting persistent activation of inflammatory and catabolic pathways within the osteoarthritic microenvironment [20,21]. Collectively, these data support the concept that osteoarthritis in the horse is characterized by sustained activation of inflammatory and proteolytic pathways within the joint, providing a biological context for interpreting the observed biomarker changes in the present study [19,22,23]. The within-subject changes observed in treated horses are consistent with alterations in interconnected inflammatory, proteolytic, and neurogenic pathways, supporting a biologically coherent pattern. Nonetheless, in the absence of longitudinal biomarker data in the comparison group, these findings should be interpreted as exploratory. The simultaneous attenuation of these interconnected pathways is biologically consistent with the proposed mechanism of broad protease inhibition and provides mechanistic plausibility for the observed clinical response. The coordinated reductions observed in inflammatory cytokines, matrix metalloproteinases, sulphated glycosaminoglycans and neurogenic mediators in the present study suggest modulation of multiple interconnected pathways involved in osteoarthritic progression [1]. Several limitations should be considered when interpreting these results. Allocation was not randomized, and group assignment was influenced by owner consent and clinical decision-making, introducing potential residual confounding. Biomarker analyses were performed exclusively in treated horses, preventing direct comparison of molecular trajectories between treated and untreated joints. An additional methodological limitation concerns the absence of repeat arthrocentesis in comparison horses. Synovial biomarker analyses were performed longitudinally only in horses receiving the investigated intra-articular orthobiologic intervention. Comparison horses were managed according to conservative clinical management protocols under field conditions and were not subjected to repeated arthrocentesis exclusively for research-related biomarker assessment. While this approach is consistent with clinical practice, it limits direct comparison of biomarker trajectories between groups and represents an inherent constraint of the study design. Furthermore, arthrocentesis itself may induce transient synovial responses associated with needle insertion and joint manipulation, introducing potential procedural effects that cannot be fully excluded. Previous studies investigating synovial biomarker dynamics in untreated or placebo-treated equine joints have reported relative short-term stability of inflammatory mediators in the absence of effective intervention [6,19,24,25]. This observation further supports the interpretation that the coordinated reductions observed in inflammatory cytokines, proteolytic enzymes and neurogenic mediators in the treated horses likely reflect biological modulation associated with the intervention rather than spontaneous fluctuations over time [26]. In addition, the statistical modeling approach, which accounted for repeated measures and baseline values, supports the presence of consistent changes from baseline over time in treated horses rather than random fluctuation. This pattern is further supported by the coherence between biomarker trends and clinical outcomes, with treated horses showing improvement in clinical parameters while minimal changes were observed in the comparison group. Overall, the internal coherence between sustained clinical improvement and coordinated modulation of inflammatory, proteolytic and neurogenic mediators strengthens the biological interpretation of these findings. The results provide a rationale for future randomized controlled trials aimed at confirming clinical efficacy and further defining the role of protease-targeted modulation in the management of equine joint disease. No safety concerns were identified during the 180-day follow-up period; however, the limited sample size does not allow definitive conclusions regarding the safety profile. The present study was not designed to establish therapeutic efficacy but to explore clinical and biological patterns associated with intra-articular alpha-2-macroglobulin administration. The observed association between clinical improvement and biomarker changes should therefore not be interpreted as evidence of a causal relationship. Instead, these findings provide preliminary signals supporting the biological plausibility of protease-targeted approaches and warrant confirmation in appropriately designed randomized controlled studies.

## 5. Conclusions

In this exploratory prospective pilot study, intra-articular alpha-2-macroglobulin therapy was associated with sustained improvement in clinical parameters in sport horses affected by chronic joint disease. In treated horses, parallel within-subject reductions were observed across inflammatory, catabolic and neurogenic synovial biomarkers, including pro-inflammatory cytokines, matrix metalloproteinases, sulphated glycosaminoglycans, NGF and Substance P. These changes are consistent with alterations in interconnected molecular pathways involved in synovial inflammation and nociceptive signaling. However, as synovial biomarker data were not collected longitudinally in the comparison group, these findings should be interpreted as exploratory and do not allow causal inference. While not demonstrating structural disease modification, the observed coherence between clinical trends and biomarker changes supports the biological plausibility of protease-targeted approaches and provides a rationale for further controlled studies in equine joint disease.

## Data Availability

The original contributions presented in this study are included in the article. Further inquiries can be directed to the corresponding author.
